# Alterations in arginine and energy metabolism, structural and signalling lipids in metastatic breast cancer in mice detected in plasma by targeted metabolomics and lipidomics

**DOI:** 10.1186/s13058-018-1075-y

**Published:** 2018-12-04

**Authors:** Kamil Kus, Agnieszka Kij, Agnieszka Zakrzewska, Agnieszka Jasztal, Marta Stojak, Maria Walczak, Stefan Chlopicki

**Affiliations:** 1 Jagiellonian University, Jagiellonian Centre for Experimental Therapeutics, Bobrzynskiego 14, 30-348 Krakow, Poland; 2 Jagiellonian University Medical College, Chair and Department of Toxicology, Medyczna 9, 30-688 Krakow, Poland; 3 Jagiellonian University Medical College, Chair of Pharmacology, Grzegorzecka 16, 31-531 Krakow, Poland

**Keywords:** Breast cancer, Metastasis, Metabolomics, Arginase, Lipids

## Abstract

**Background:**

The early detection of metastasis based on biomarkers in plasma may improve cancer prognosis and guide treatment. The aim of this work was to characterize alterations in metabolites of the arginine pathway, energy metabolism, and structural and signalling lipids in plasma in the early and late stages of murine breast cancer metastasis.

**Methods:**

Mice were orthotopically inoculated with 4T1 metastatic breast cancer cells, and plasma was analysed along the pulmonary metastasis progression using LC-MS/MS-based targeted metabolomics and lipidomics.

**Results:**

Based on primary tumour growth and pulmonary metastases, 1–2 weeks after 4T1 cancer cell inoculation was defined as an early metastatic stage, and 3–4 weeks after 4T1 cancer cell inoculation was defined as a late metastatic stage. Early metastasis was featured in plasma by a shift of L-arginine metabolism towards arginase (increased ornithine/arginine ratio) and polyamine synthesis (increased putrescine). Late metastasis was reflected in plasma by further progression of changes in the arginine pathway with an additional increase in asymmetric dimethylarginine plasma concentration, as well as by a profound energy metabolism reprogramming towards glycolysis, an accelerated pentose phosphate pathway and a concomitant decrease in tricarboxylic cycle rate (“Warburg effect”). The late but not the early phase of metastasis was also characterized by a different lipid profile pattern in plasma, including a decrease in total phosphatidylcholines, a decrease in diester-bound phospholipid fraction and an increase in lysophospholipids associated with an increase in total sphingomyelins.

**Conclusions:**

The early phase of metastasis in murine 4T1 metastatic breast cancer was associated with plasma metabolome changes characteristic of arginase activation and polyamine synthesis. The late metastasis was reflected in plasma not only by the alterations in arginine pathways but also by a shift towards glycolysis and the pentose pathway, remodelling of structural lipids and activation of lipid signalling, all of which coincided with metastasis progression.

**Electronic supplementary material:**

The online version of this article (10.1186/s13058-018-1075-y) contains supplementary material, which is available to authorized users.

## Introduction

Although significant progress has been made in cancer research during recent decades, breast cancer is still the most frequently diagnosed tumour in women worldwide. The survival rate of primary breast cancer is now close to 100%, but it decreases to only 25% once distant metastasis has occurred [1]. The prognosis and patient survival depend on the cancer stage at the time of diagnosis. Therefore, dozens of studies have been conducted with the aim of developing new biomarkers that could be used for screening, diagnosis and prognosis of cancer. Metabolomics, as the endpoint of the ‘omics’ cascade, is focused on the investigation of the global metabolites present in a biological specimen and is considered representative of the phenotype [2]. Recent metabolomic studies have expanded knowledge of the mechanisms underlying cancer pathogenesis [3], and several reports in the field of biomarker research have been published using untargeted approaches that have enabled the measurement of thousands of metabolites with the goal of detecting previously unpredicted metabolite perturbations. On the other hand, targeted studies, by quantitative measurements of relatively smaller but pathway-oriented metabolites, enable tracking the changes in a vast array of metabolic enzymes, kinetics and end-products of a given pathway known or suspected to be relevant to the disease pathophysiology [4].

Several recent publications have demonstrated that targeted metabolomics can prove useful not only for diagnosis and monitoring response to treatment but also for finding a relationship between clinicopathological characteristics of breast cancer and various metabolites in blood, urine and tissue specimens [5]. Depletion of the arginine plasma pool, reprogramming of glucose metabolism and changes in lipid metabolism reflecting either the increased arginine and glucose use during cancer progression, hypoxic metabolism or restructuring of the cell membranes in tumours [6] are important and pathophysiologically relevant changes that accompany tumour development and may influence cancer prognosis.

Arginine is a dibasic, semi-essential amino acid involved in many biosynthetic pathways that can significantly influence carcinogenesis and tumour biology. It is metabolized mainly by arginase (ARG) to urea and ornithine, which can be further transformed into polyamines, and by nitric oxide synthase (NOS) into nitric oxide (NO) and citrulline [7]. Polyamines are involved in cellular proliferation [8], whereas metabolism of L-arginine by ARG was found to be an important factor in controlling the immune response, suppressing anti-tumour immune response and promoting tumourigenesis [9, 10]. In particular, myeloid-derived suppressor cells (MDSCs) consisting of precursors of granulocytes, macrophages or dendritic cells that potently inhibit the maturation and function of lymphocytes T, natural killer and dendritic cells were reported to greatly expand in human [11–13] and murine cancer [14], and their activity was linked to increased arginine metabolism by ARG and inducible nitric oxide synthase (iNOS) [15].

Metabolism reprogramming towards aerobic glycolysis—the so-called ‘Warburg Effect’ [16]—represents an important adaptation of cancer cells to support cell survival, tumour growth, tissue remodelling and cancer metastasis, and it has been established as a new hallmark of cancer.

Finally, changes in lipid metabolism were shown to be closely associated with cancer progression. Alterations in lipid composition and abundance have been established as a hallmark of cancer aggressiveness [17] that may strongly influence both physical properties of membranes, such as bilayer thickness, lipid packing density, membrane fluidity or surface charge [18], and the signal transduction process upon conversion into bioactive lipid mediators.

Given that changes in arginine metabolism [19–21], energy metabolism reprogramming [21–23] and structural and signalling lipids [23–27] have been described as potential biomarkers of cancer detection, progression and prognosis, our goal was to find the earliest changes in these pathways in plasma along the development of breast cancer progression. We used a murine 4T1 carcinoma model because of its predisposition to spontaneous metastasis to the lung [28] using a low number of inoculated cells, resulting in a relatively protracted time course of pulmonary metastasis development, as observed in our previous study [29–32]. We attempted to perform targeted metabolomic analysis in blood plasma, not locally in the tumour tissue, to evaluate measurable systemic changes with potentially diagnostic significance for the early phase of metastasis development.

We identified that the first systemically detectable metabolic changes in plasma were linked with a shift in arginine metabolism toward ARG, associated with early changes in immune response as it coincided with infiltration of granulocytes and extramedullary haematopoiesis. Later changes in arginine metabolism also involved an increase in plasma asymmetric dimethylarginine (ADMA) concentration, suggesting endothelial dysfunction. In turn, the late phase of metastasis was associated with plasma changes suggesting energy metabolism reprogramming, modulation of structural lipid content, and increased release of pro-inflammatory lipid mediators, all of which coincided with tumour growth and metastasis formation.

## Methods

### Murine model of metastatic breast cancer

Seven- to eight-week-old BALB/c female mice (obtained from the Centre of Experimental Medicine, Medical University of Bialystok, Poland) were orthotopically inoculated into the right mammary fat pad with 1 × 10^4^ viable mouse mammary adenocarcinoma 4T1 cells (American Type Culture Collection, Manassas, VA, USA) cultured according to a previously described protocol [29]. Healthy BALB/c mice injected with Hanks’ balanced salt solution without 4T1 cells were used as a control group. The overall number of mice in the tumour-bearing group consisted of 40 mice (10 mice per each group 1, 2, 3 and 4 weeks after cancer cell inoculation). The control group consisted of 40 mice (10 mice per each group 1, 2, 3 and 4 weeks after Hanks’ balanced salt solution injection). At each time point, the groups of 10 tumour-bearing mice and 10 respective control mice were injected with intraperitoneal (i.p.) administration of heparin (25 IU/mouse) and anaesthetized by an i.p. injection of ketamine and xylazine (100 mg ketamine/10 mg xylazine/kg body weight). Mice were killed, then their blood was drawn by cardiac puncture, and their internal organs were perfused in situ with Krebs buffer until blood-free, dissected, weighed and collected for further analysis.

All experimental procedures involving animals were conducted according to the Guidelines for Animal Care and Treatment of the European Communities and the Guide for the Care and Use of Laboratory Animals of the National Institutes of Health. All procedures were approved by the Local Ethical Committee on Animal Experiments at the Jagiellonian University (17/2016).

Blood count was measured by using an ABC Vet analyser (scil Animal Care Co., Gurnee, IL, USA), and remaining blood was centrifuged at 3000 × *g* at 4 °C for 12 min, with plasma then kept for further analysis. Tumour size was calculated by measuring the tumour length (L) and width (W) using an electronic calliper and expressed as tumour volume *V* = 0.52 × *L* × *W*^2^. In order to determine pulmonary metastasis, representative lung tissues from four mice were kept in buffered formalin solution, and the macroscopic lesions were counted in the lungs, as previously described [29]. For examination of liver haematopoiesis by histological analysis, standard H&E staining of paraffin-embedded liver tissue was used. Stained sections were examined and photographed at 100× magnification with an Olympus BX51 light microscope (Olympus Corporation, Tokyo, Japan).

### Determination of metabolites from arginine metabolism, energy metabolism and lipid content in plasma

In order to determine the concentration of selected metabolites from studied pathways, three LC-MS/MS targeted metabolomics-based methods were applied. The arginine metabolites were evaluated on the basis of LC-MS/MS measurements using the AbsoluteIDQ p180 Kit (Biocrates Life Sciences, Innsbruck, Austria) and a developed LC-MS/MS targeted-metabolomic method, which was also used for energy metabolite determination. The lipid profile was evaluated using a flow injection analysis (FIA)-MS/MS approach from the AbsoluteIDQ p180 Kit. The concentration of sphingosine-1-phosphate (S1P) was measured by using an LC-MS/MS-based method, described below.

Energy metabolites (e.g., intermediates of glycolysis, tricarboxylic acid cycle [TCA], pentose phosphate pathway (PPP), amino acids and other intracellular metabolites measured in plasma) were measured by employing an LC-MS/MS technique on a QTRAP 5500 mass spectrometer (SCIEX, Framingham, MA, USA) coupled to a UFLC Nexera chromatograph (Shimadzu, Kyoto, Japan) using an Acquity UPLC BEH C18 column, 1.7 μm, 3.0 × 100 mm (Waters, Milford, MA, USA) as an analytical column. Samples were analysed twice in positive and negative ionization multiple reaction monitoring mode. The detailed methodology is included in Additional file 1.

Amino acids, biogenic amines and lipids in plasma were measured using the commercially available AbsoluteIDQ p180 kit, which allows for simultaneous quantification of 21 amino acids, 14 biogenic amines, 40 acylcarnitines, 90 glycerophospholipids, 15 sphingolipids and sum of hexoses. The sample preparation and measurement were carried out according to the kit manufacturer’s instructions. Briefly, 10 μl of standards, quality controls and plasma samples were pipetted onto filter paper within each well plate of the 96-well plate provided by the kit manufacturer. The samples were dried under nitrogen, followed by application of phenylisothiocyanate (MilliporeSigma, Burlington, MA, USA), then they were dried again, and metabolites were extracted from the filter paper using 5 mM ammonium acetate in methanol solution and diluted. Samples were measured twice by LC-MS/MS (amino acid and biogenic amines) and FIA-MS/MS (lipids and hexoses) using the QTRAP 5500 mass spectrometer coupled to a UFLC Nexera chromatograph. Quantification of metabolite concentration and quality assessment were performed with MetIQ software (Biocrates Life Sciences, Innsbruck, Austria).

The concentration of S1P in plasma was determined by using an LC-MS/MS technique with a TSQ Quantum mass spectrometer system (Thermo Scientific, Waltham, MA, USA) coupled to a UHPLC UltiMate 3000 (Dionex, Sunnyvale, CA, USA). Chromatographic separation was achieved with a Hypersil GOLD analytical column (1.9 μm, 2.1 mm × 50 mm; Thermo Scientific, Waltham, MA, USQ). The detailed methodology is included in Additional file 1.

### Statistical analysis

Statistical analysis was performed using STATISTICA 12 software (StatSoft Inc., Tulsa, OK, USA). All values are expressed as mean ± SEM. The assessment of normality and homogeneity of variances was performed using the Shapiro-Wilk and Levene tests, respectively. To assess the statistical significance, one-way analysis of variance (ANOVA), Tukey’s posthoc test and a non-parametric Kruskal-Wallis ANOVA were performed. *P* values are provided in the figure legends. For multivariate analysis, data of all experiments were merged into a matrix table. Partial least squares discriminant analysis (PLS-DA) and heat map clustering of the data were performed using web-based software (MetaboAnalyst 4.0) [33].

## Results

### Primary tumour growth and pulmonary metastasis

**Fig. 1:**
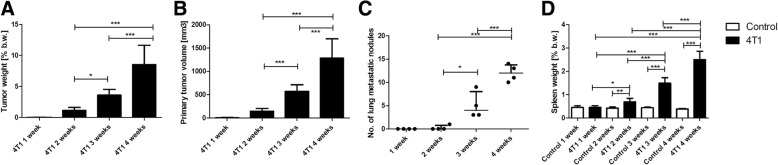
Primary tumour growth and development of pulmonary metastasis in a murine model of 4T1 metastatic breast cancer. Primary tumour growth: weight (**a**) and volume (**b**) of primary tumour. Lung metastasis is expressed as the number of metastatic nodules (**c**). Empty bars represent control animals, and black bars represent tumour-bearing mice. Occurrence of systemic inflammation is expressed as the increase of spleen weight (**d**). Data are expressed as mean ± SEM (*n* = 10 for organ weight and *n* = 4 for lung metastatic nodules). Based on the normality of distribution and variance homogeneity (*F* test), data were analysed by analysis of variance followed by Tukey’s post hoc test with statistical significance set at *p < 0.05, **p < 0.01, ***p < 0.001

The primary tumour was barely palpable 1 week after 4T1 breast cancer cell inoculation, and reliable measurement of primary tumour was possible starting from 2 weeks after 4T1 breast cancer cell inoculation. Two weeks after 4T1 cancer cell inoculation, the mean tumour weight and volume were 0.22 ± 0.08 g and 149.6 ± 57.8 mm^3^, respectively, which represented c.a. 1.18% of body weight, whereas 4 weeks after 4T1 cell inoculation, the mean tumour weight and volume were 1.85 g ± 0.65 g and 1285 ± 415 mm^3^, respectively, which represented 10% of total body weight (Fig. 1a, b).

The pulmonary metastasis was not observed 1 week after 4T1 breast cancer cell inoculation, and only a single metastatic nodule in the lungs was observed 2 weeks after cell inoculation. Three and four weeks after cancer cell inoculation, metastatic lung nodules were present in all tumour-bearing mice, reaching c.a. 5 and 12 metastatic nodules, respectively (Fig. 1c).

No difference in spleen weight, an indirect parameter of systemic inflammatory state [29], was found 1 week after cancer cell inoculation, and a slight increase in spleen weight was observed 2 weeks after 4T1 cell inoculation (0.134 ± 0.028 g vs 0.07 ± 0.006 g in control mice), whereas it was markedly increased 3 weeks (0.295 ± 0.057 g) and 4 weeks (0.52 ± 0.08 g) after cancer cell inoculation (Fig. 1d). These results were consistent with the changes in blood count. The number of circulating white blood cells was elevated from 2 weeks after 4T1 cell inoculation (12.8 ± 6.9 × 10^3^/μl vs 4.1 ± 0.7 × 10^3^/μl in control mice) and reached 50.6 ± 15.89 × 10^3^/μl and 189.9 ± 73.7 × 10^3^/μl 3 and 4 weeks, respectively, after cancer cell inoculation. Cancer-associated leucocytosis was linked with a relative increase in number of granulocytes and monocytes and a decreased number of lymphocytes, which was pronounced 3–4 weeks after cancer induction (Table 1).

**Table 1:** Circulating white blood cell analysis^a^

	1 week		2 weeks		3 weeks		4 weeks	
	Control	4T1	Control	4T1	Control	4T1	Control	4T1
WBC^b^	4.5 ± 1.1	4.3 ± 1.5	4.1 ± 0.7	12.8 ± 6.9	4.8 ± 0.8	50.6 ± 15.9	4.4 ± 1.1	189.0 ± 73.7
GRA^c^	1.1 ± 0.2	1.4 ± 0.5	1.0 ± 0.3	8.1 ± 5.5	1.0 ± 0.2	35.7 ± 12.7	0.9 ± 0.3	143.6 ± 55.2
GRA%^d^	23.6 ± 7.2	31.5 ± 6.8	21.1 ± 4.0	57.8 ± 11.6	18.6 ± 1.6	69.7 ± 4.9	18.4 ± 2.9	75.0 ± 3.6
LYM^c^	3.3 ± 1.0	2.7 ± 1.0	3.0 ± 0.5	4.1 ± 1.2	3.7 ± 0.7	11.5 ± 2.8	3.3 ± 0.8	31.5 ± 11.9
LYM%^d^	71.8 ± 8.9	63.5 ± 7.4	75.1 ± 4.5	36.9 ± 11.2	77.6 ± 1.6	23.5 ± 3.8	77.6 ± 3.0	17.2 ± 2.4
EOS^c^	3.6 ± 1.4	5.0 ± 1.1	3.3 ± 1.1	11.4 ± 4.4	3.5 ± 0.6	9.2 ± 3.4	3.0 ± 1.7	66.5 ± 13.7
MON^c^	0.14 ± 0.1	0.2 ± 0.1	0.1 ± 0.02	0.6 ± 0.3	0.1 ± 0.03	3.4 ± 1.4	0.1 ± 0.05	14.9 ± 8.3
MON%^d^	4.6 ± 1.9	4.8 ± 1	3.8 ± 0.5	5.3 ± 1.1	3.8 ± 0.4	6.8 ± 1.8	3.9 ± 0.4	7.8 ± 1.8

*Abbreviations: EOS* Eosinophils, *GRA* Granulocytes, *LYM* Lymphocytes, *MON* Monocytes, *WBC* White blood cells

^a^Whole blood was collected by cardiac puncture at the indicated time after orthotopic introduction of tumour cells, as described in the Methods section of text

^b^Total number of WBC per microliter of blood in thousands ± SEM

^c^Number of cells per microliter of blood in thousands ± SEM

^d^The percentage of total WBC ± SEM

Altogether, based on primary tumour growth and progression of metastases to the lung and spleen weight, the 1–2-week period after 4T1 cancer cell inoculation was defined as pre-metastatic and early metastatic stages with a relatively small primary tumour, scarce pulmonary metastasis and early stage of systemic inflammation, whereas the 3–4 week period after 4T1 cancer cell inoculation was considered the late metastatic stage with accelerated primary tumour growth, advanced pulmonary metastasis and severe systemic inflammation.

### Alterations in arginine metabolism

**Fig. 2:**
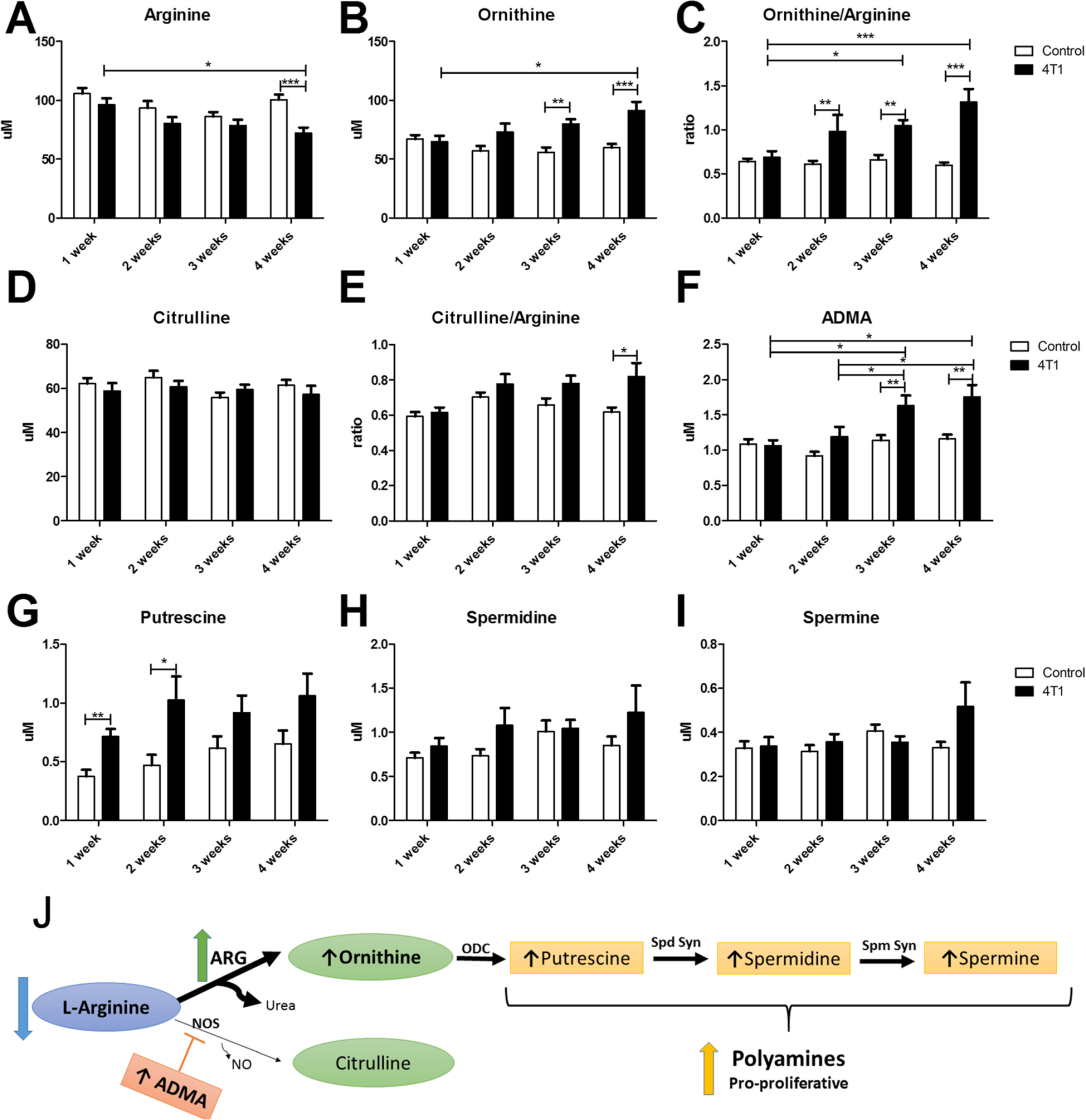
Shunts of arginine metabolism into arginase pathway, synthesis of polyamines and asymmetric dimethylarginine (ADMA) detected in plasma in murine model of 4T1 metastatic breast cancer. Decrease in arginine plasma pool (**a**), increase in ornithine concentration (**b**) and ornithine-to-arginine ratio (**c**) in tumour-bearing mice, suggesting an increase in arginase activity. Lack of changes in citrulline plasma concentration (**d**), slight increased citrulline-to-arginine ratio (**e**) and increase in concentration of asymmetric dimethylarginine (**f**) in tumour-bearing mice, suggesting impaired nitric oxide synthase (NOS) activity. Increased plasma concentrations of putrescine (**g**), spermidine (**h**) and spermine (**i**) in tumour-bearing mice suggesting increased polyamine synthesis. An overview of 4T1 breast cancer cell-related alterations in L-arginine metabolism (**j**). Empty bars represent control animals, and black bars represent tumour-bearing mice. Data are expressed as mean ± SEM (*n* = 10). The data were analysed with either analysis of variance (ANOVA) followed by Tukey’s post hoc test (arginine, ornithine/arginine, putrescine) or non-parametric Kruskal-Wallis ANOVA (ornithine, citrulline, citrulline/ornithine, ADMA, spermidine, spermine), depending on the normality of distribution and variance homogeneity (*F* test) with statistical significance set at *p < 0.05, **p < 0.01, ***p < 0.001. *ARG* Arginase, *ODC* Ornithine decarboxylase, *Spd Syn* Spermidine synthase, *Spm Syn* Spermine synthase

The arginine plasma concentration tended to decrease in early metastatic stage, but the difference reached statistical significance 4 weeks after 4T1 cancer cell inoculation, suggesting increased arginine use in tumour-bearing mice (Fig. 2a). The plasma ornithine concentration increased significantly 3 weeks after 4T1 cancer cell inoculation (Fig. 2b). Overall, the ornithine-to-arginine ratio, compatible with the activation of ARG that catalyses the catabolism of arginine to ornithine and urea, was significantly increased as early as 2 weeks after 4T1 cancer cell inoculation (Fig. 2c). In the early phase of metastasis ornithine-derived pro-proliferative polyamines—putrescine, spermidine and spermine (Fig. 2g–i)—also increased, with most pronounced early changes in plasma concentration of putrescine (2 weeks after cancer cell inoculation). The aforementioned changes in arginine metabolism were even more pronounced in the late phase of metastasis.

In turn, the plasma concentration of citrulline, the NOS-derived metabolite of arginine, did not change significantly along breast cancer development (Fig. 2d). The citrulline-to-arginine ratio increased only slightly (Fig. 2e), but it was related to arginine decrease rather than to citrulline increase and was visible only in the late phase of metastasis (4 weeks after cancer cell inoculation). Endogenous inhibitor of NOS—ADMA—substantially increased (Fig. 2f) only in the late phase of metastasis (3–4 weeks after cancer cell inoculation), suggesting decreased NOS activity in the late metastatic phase of breast cancer progression.

**Fig. 3:**
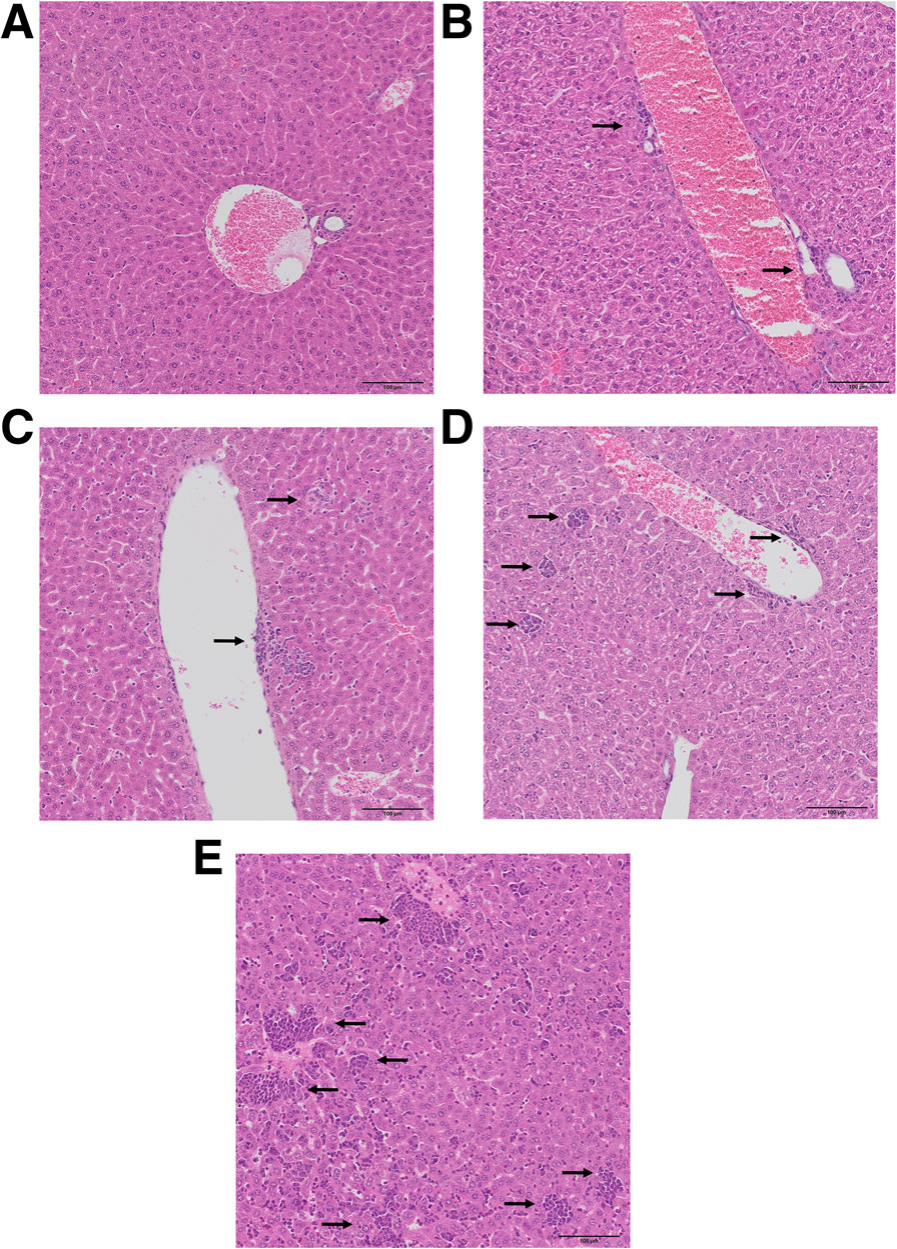
Extensive extramedullary haematopoiesis in the liver during cancer progression in a murine model of 4T1 metastatic breast cancer. Normal liver in the control group (**a**). The progressive increase in the area of hematopoietic centres in the liver during 4T1 metastatic breast cancer progression (**b**–**e**). Small island of extramedullary haematopoiesis in the vicinity of blood vessel walls (first week after 4T1 cell inoculation) (**b**); increase of hematopoietic centre size near blood vessels and formation of new foci in the liver parenchyma, visible features of proliferation and initial stages of cell differentiation (second week after 4T1 cell inoculation) (**c**); formation of numerous hematopoietic centres in liver tissue, both in the vicinity of vascular walls and in liver parenchyma, visible cells in different and late stages of differentiation (third week after 4T1 cell inoculation) (**d**); further increase in number and size of hematopoietic centres, visible variation in the type of produced leucocytes, in the winded sinusoids, numerous leucocytes, among which also immature leucocytes are visible (fourth week after 4T1 cell inoculstion) (**e**)

In order to correlate the changes in arginine metabolisms in plasma with the activation of extramedullary haematopoiesis, the latter reported previously to occur in this model [28, 34, 35], we analysed liver samples by histology. As shown in Fig. 3, small centres of extramedullary haematopoiesis with visible features of proliferation and initial stages of hematopoietic cell differentiation were detected in the liver as early as 1–2 weeks after cancer cell inoculation (Fig. 3). The size and number of hematopoietic centres increased substantially in the late metastatic stage, 3–4 weeks after cancer induction, with extensive proliferation and late phases of differentiation. Moreover, in the late stage an expansion of the number of leucocytes, among those also immature ones, was seen in enlarged (widened) liver sinusoids and conduit blood vessels within the liver (Fig. 3).

### Reprogramming of energy metabolism

**Fig. 4:**
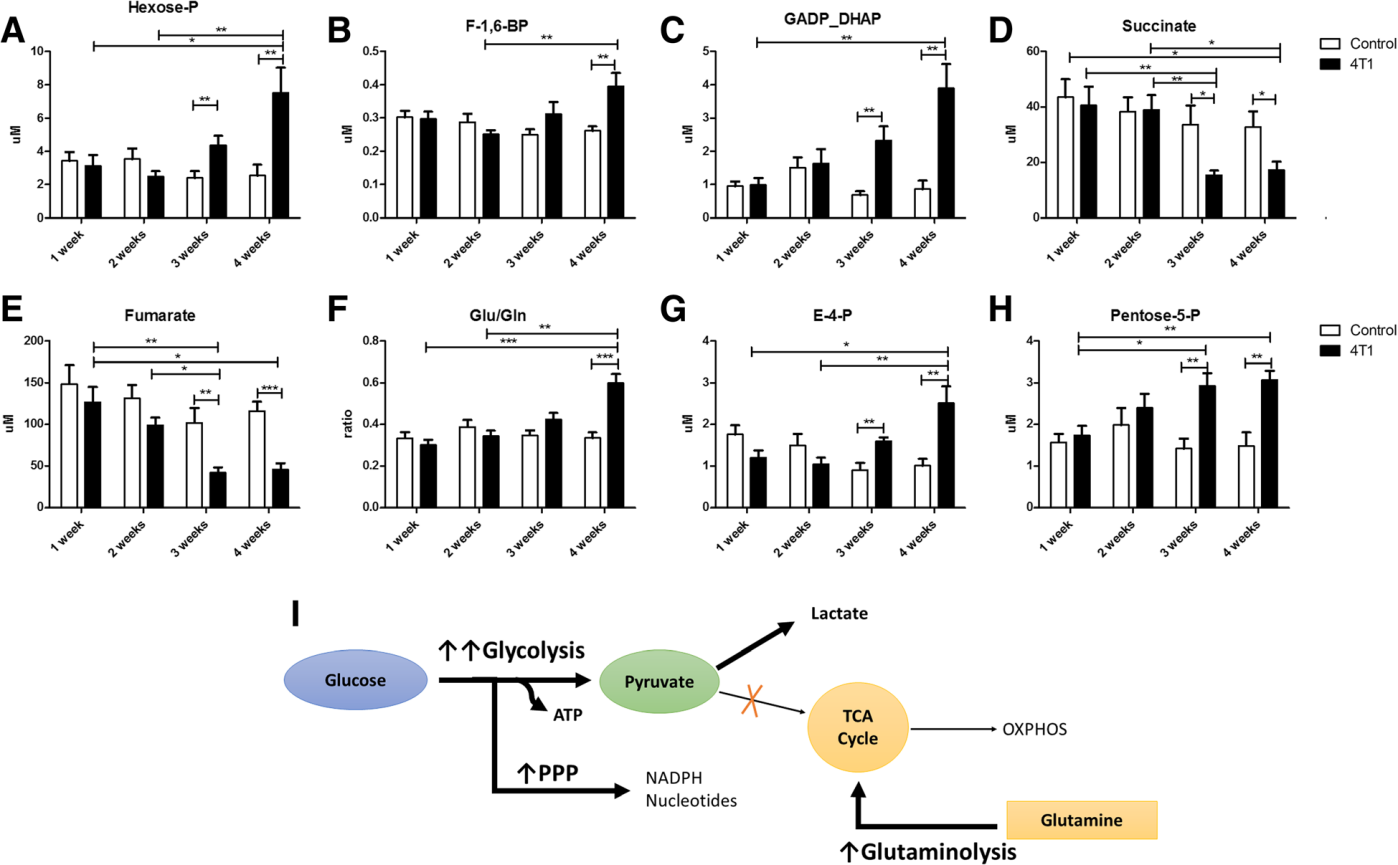
Reprogramming of energy metabolism associated with cancer growth detected in plasma in a murine model of 4T1 metastatic breast cancer. Increase in glycolytic rate, evidenced as an increase of hexose phosphate (**a**), fructose 1,6-bisphosphate (**b**), glyceraldehyde 3-phosphate and dihydroxyacetone phosphate (**c**) concentrations measured in plasma, and a decrease of tricarboxylic acid cycle intermediate plasma concentrations of succinate (**d**) and fumarate (**e**). Increase of glutamine catabolism evidenced as an increase in glutamate-to-glutamine ratio (**f**) and acceleration of pentose phosphate pathway increase of erythrose 4-phosphate (**g**) and pentose phosphate concentrations (**h**). A simplified overview of 4T1 breast cancer-induced changes in energy metabolism (**i**). Empty bars represent control animals, and black bars represent tumour-bearing mice. Data are expressed as mean ± SEM (n = 10). The data were analysed with either analysis of variance (ANOVA) followed by Tukey’s post hoc test (GADP_DHAP, fumarate, Gln/Glu) or a non-parametric Kruskal-Wallis ANOVA (Hexose-P, fructose 1,6-bisphosphate, succinate, E4P, Pentose-5P), depending on the normality of distribution and variance homogeneity (*F* test), with statistical significance set at *p < 0.05, **p < 0.01, ***p < 0.001

As shown in Fig. 4, the late phase of metastasis (3–4 weeks after 4T1 cell inoculation) but not the early phase was associated with activation of glycolysis, pentose phosphate pathway, glutaminolysis and diminution of the TCA cycle, as evidenced by altered plasma concentrations of the respective metabolites. The concentrations of glycolytic intermediates measured in plasma were elevated (e.g., concentration of hexose phosphate and triose phosphate [dihydroxyacetone phosphate {DHAP}] and D-glyceraldehyde-3-phosphate) were increased by 50% and 200%, respectively, 3 weeks after 4T1 cell inoculation and by approximately 150% and 400%, respectively, 4 weeks after 4T1 cell inoculation as compared with the control mice (Fig. 4a–c). Concomitant with an increase in glycolytic intermediates, the plasma levels of TCA products (e.g., fumarate and succinate) decreased 3–4 weeks after cancer cell inoculation (Fig. 4d, e). Moreover, the increase of glutamine (Gln) catabolism, evidenced as increased glutamate to glutamine ratio (Glu/Gln) and acceleration of the PPP (increased erythrose-4-phosphate [E-4-P] and pentose-5-phosphate [Pentose-5-P])(was observed only at the late and not at the early phase of metastasis (Fig. 4f–h).

### Alterations in structural and signalling lipids

**Fig. 5:**
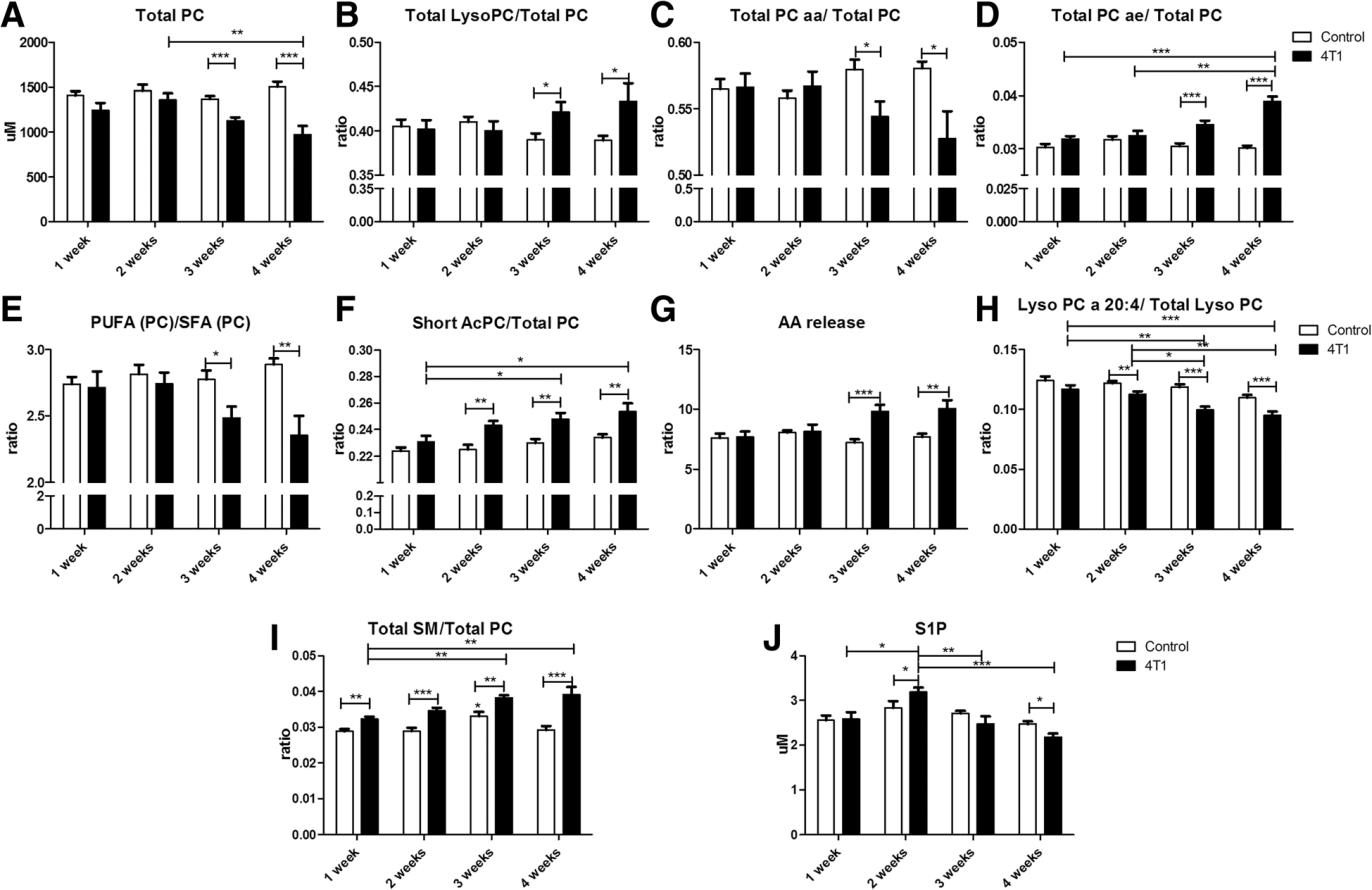
Changes in lipid fractions detected in plasma in a murine model of 4T1 metastatic breast cancer indicating alterations in membrane structure and lipid signalling. Increase of phospholipase A2 activity observed as a decrease in total plasma phosphatidylcholines (**a**) and an increase of lysophosphatidylocholines (**b**). Changes in diacyl chain phosphatidylcholine fractions: decrease in diester-bound phosphatidylcholines (**c**) and increase in ester-ether-bound (plasmalogen) fraction (**d**). Decrease in unsaturation of plasma lipids, measured as a ratio of polyunsaturated lipids to saturated lipids (**e**). Increased short-chain phospholipid fraction (**f**). Increase in arachidonic acid release, measured indirectly as a sum of specific ratios of most abundant lysophosphatidylocholines to phosphatidylcholines indicating release of arachidonic acid (LysoPC 16:0/PC 36:4, LysoPC 16:1/PC 36:1, LysoPC 18:0/ PC 38:4, LysoPC 18:1/PC 38:5, LysoPC18:2/PC 38:6) (**g**) and decrease of lysophosphatidylocholine-containing arachidonic acid (**h**). Increase of total amount of sphingomyelins (SMs) in 4T1 tumour-bearing mice (**i**). Changes in the plasma concentration of sphingosine-1-phosphate (**j**). Empty bars represent control animals, and black bars represent tumour-bearing mice. Data are expressed as mean ± SEM (n = 10). The data were analysed with either analysis of variance (ANOVA) followed by Tukey’s post hoc test (Total PC ae/Total PC, Lyso PC a 20:4/Total Lyso PC, Total SM/Total PC) or a non-parametric Kruskal-Wallis ANOVA (Total PC, Total Lyso PC/Total PC, Total PC aa/Total PC, PUFA (PC)/SFA (PC), Short AcPC/Total PC, AA release, S-1-P), depending on the normality of distribution and variance homogeneity (*F* test), with statistical significance set at *p < 0.05, **p < 0.01, ***p < 0.001

The main changes in plasma phosphatidylcholine profile were observed at the late phase of metastasis (3–4 weeks after cancer cell inoculation) and included a decrease in total phosphatidylcholine level (Total PC) with a simultaneous increase in the concentration of lysophosphatidylocholines (LysoPC), the changes being compatible with the increase in phospholipase A2 activity (Fig. 5a, b). The profile of phosphatidylcholines was also altered, because the fraction of phosphatidylcholines having two acyl chains attached by ester linkage decreased, whereas lipids that contain one acyl chain attached by an ether bound increased (Fig. 5c, d). Additionally, 3–4 weeks after cancer cell inoculation the unsaturation of acyl chains in phosphatidylcholines decreased, as evidenced by the reduction of the ratio of polyunsaturated phosphatidylcholines to saturated phosphatidylcholines by c.a. 20% (Fig. 5e) and shortening of the acyl-chain length in phosphatidylcholine structures (Fig. 5f). The release of arachidonic acid from phosphatidylcholines was augmented at the late phase but not the early phase of metastasis, as evidenced by the increase in the ratio of various LysoPC to phosphatidylcholines indicating release of 20:4 fatty acid arachidonate (LysoPC 16:0/PC 36:4; LysoPC 16:1/ PC 36:1; LysoPC 18:0/ PC 38:4; LysoPC 18:1/PC 38:5; LysoPC18:2/PC 38:6) and decrease of LysoPC containing arachidonic acid (LysoPC 20:4) (Fig. 5g, h).

The decrease in PC fraction was related to a concomitant increase in sphingomyelin (SM) fraction in the plasma phospholipid profile, also visible to some extent in the early phase of metastasis. The SM content was elevated by c.a. 15% and 30% at 2 and 4 weeks, respectively, after cancer cell inoculation (Fig. 5i). The changes in concentration of S1P, a SM-derived signalling molecule, displayed a biphasic course, being increased at the early phase of metastasis (2 weeks after cancer cell inoculation) and then significantly reduced in the late phase of metastasis (Fig. 5j).

### Partial least squares discriminant analysis

**Fig. 6:**
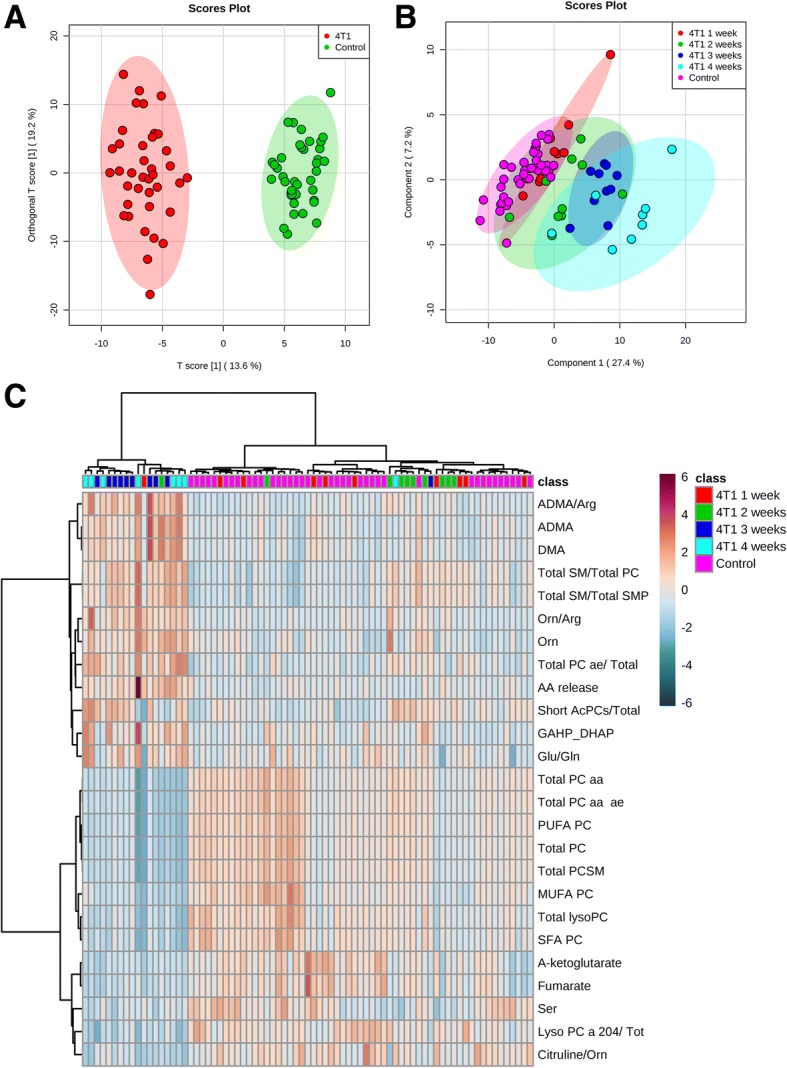
Changes of the measured metabolites in plasma of control and tumour-bearing mice. Orthogonal partial least squares discriminant analysis score plot of the qualified metabolites in plasma of control and tumour-bearing mice showing classes separation according to their metabolic signature (**a**). Sparse partial least squares discriminant analysis score plot to differentiate control and tumour-bearing mice 1–4 weeks after cancer cell inoculation (**b**). Heat map and hierarchical clustering of discriminant metabolites in control and tumour-bearing mice 1–4 weeks after cancer cell inoculation shows differing concentrations of 25 top statistically significant metabolites in studied groups. Each column represents the average metabolite concentration of ten mice for the experimental condition (**c**)

In order to confirm the significance of changes in metabolites of the arginine pathway, energy metabolism, and structural and signalling lipids in plasma during the development of metastatic 4T1 breast cancer in mice, a multivariate statistical analysis was performed. An orthogonal PLS-DA model (Fig. 6a) that was built based on measured metabolites demonstrated robust group separation between cancer-bearing mice and control mice with R2 and Q2 of 0.712 and 0.45, respectively. Additionally, by employing sparse PLS-DA, also differentiation between control mice and mice at 1, 2, 3 and 4 weeks after cancer cell inoculation can be seen; yet, the R2 (0.577) and Q2 (0.041) values, calculated based on a ten-fold cross-validation approach were low, probably due to the small number of animals in each group (*n* = 40 for control and *n* = 10 for tumour-bearing mice 1, 2, 3 and 4 weeks after 4T1 cancer cell inoculation) (Fig. 6b). Hierarchical cluster analysis for the measured metabolites was described by the Ward algorithm and arranged according to similarity in metabolite profile. As shown in Fig. 6c, a heat map of 25 top metabolites demonstrated clear-cut separation between two main clusters: a late metastatic group (3 and 4 weeks after 4T1 cancer cell inoculation) and a control and early metastatic group (1–2 weeks after 4T1 cancer cell inoculation) together. In fact, the biggest difference can be seen between the control and late metastatic groups based on changes in arginine, energy pathways and lipids. The separation between the control and early metastatic groups, although visible, was weaker and was linked to changes in arginine metabolism and some lipids.

## Discussion

**Fig. 7:**
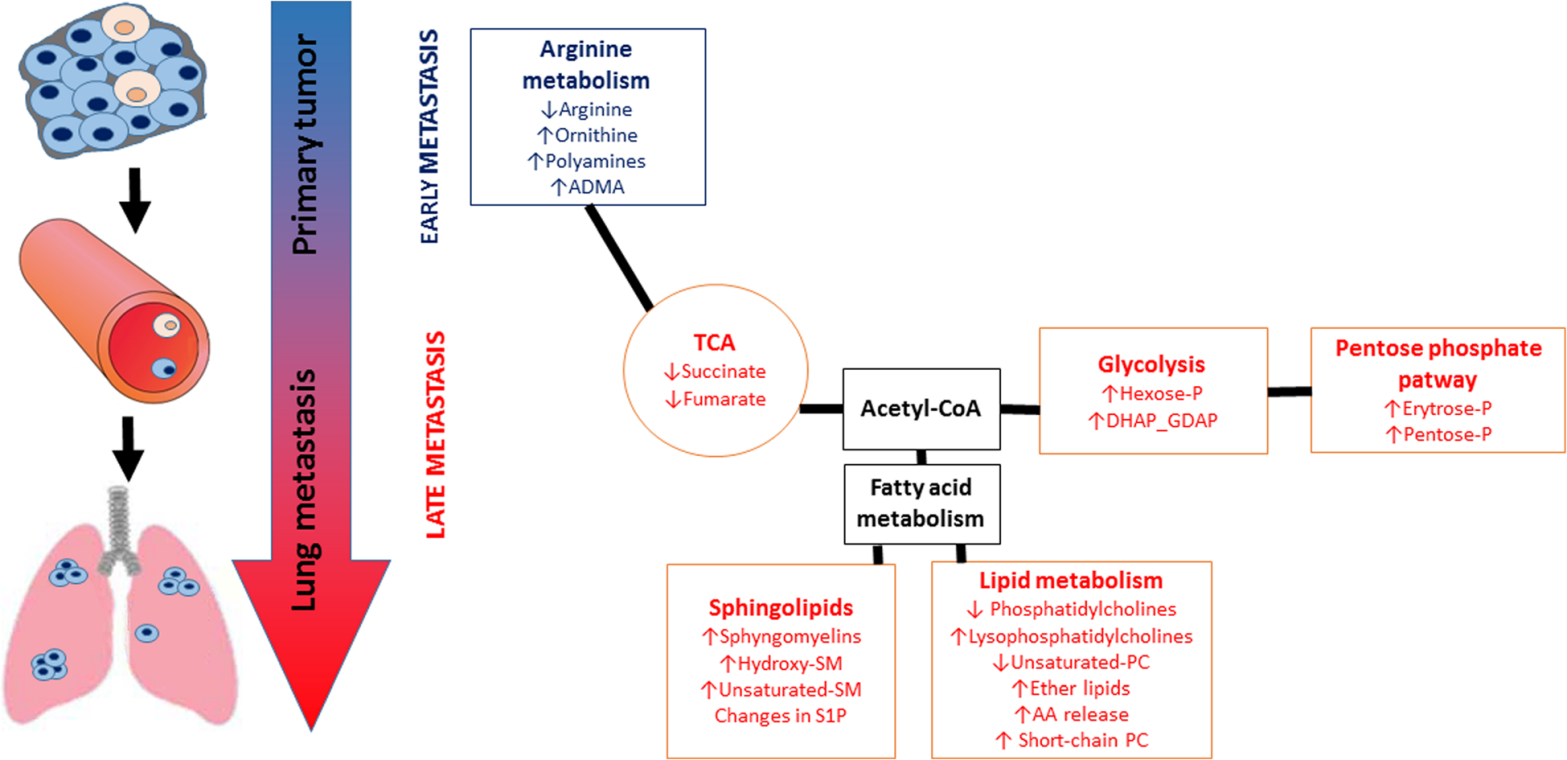
Scheme of metabolic network changes in the early and late stages of metastatic 4T1 breast cancer in mice. Early phase of metastasis is featured by a shift in L-arginine metabolism towards arginase linked with elevated polyamine biosynthesis, whereas the late phase of metastasis is characterized by energy metabolism reprogramming (‘Warburg effect’) and alterations in structural and signalling lipid profile in murine plasma

In the present work, taking advantage of targeted LC-MS-based metabolomics and lipidomics focused on arginine metabolism, energy metabolism and structural and signalling lipids, we demonstrated that in 4T1 metastatic breast cancer in mice, the early phase of metastasis was mirrored in plasma by changes in arginine metabolism characteristics for ARG activation and polyamine synthesis. The late metastasis, apart from alterations in arginine pathways, was characterized by a shift of energy metabolism towards glycolysis and PPP, remodelling of structural lipids and activation of lipid signalling (Fig. 7). Altogether, we detected changes in the plasma metabolome featuring an early phase of metastasis compatible with the shift in arginine metabolism towards ARG that could constitute an early plasma biomarker of cancer progression and metastasis and an early target of metastasis treatment [36]. In turn, a number of other changes that have been identified in energy metabolism and lipids may provide biomarkers of an advanced stage of metastatic cancer [37]*.*

The salient finding of this study was the early detection of a significant change in arginine metabolism that was detected in plasma even before the first breast cancer metastasis to the lung and involved acceleration of arginine catabolic rate as well as an increased polyamine synthesis. Acceleration of ARG activity seemed to coincide with the increased production of leukocytes (Table 1), which were identified by Donkor et al. [38] in a 4T1 model as arginine-dependent MDSCs. These MDSCs’ immunosuppressive activity, related to increased arginine metabolism via ARG and iNOS [15, 39], was shown to accompany tumour progression and metastasis [40] and caused profound perturbation in myelopoiesis, including extramedullary myelopoiesis to meet the increased demand for myeloid cell proliferation [41, 42]. Indeed, we observed an increase in the number and size of hematopoietic centres in the liver (Fig. 3), which supports active MDSC formation. Moreover, ARG activation resulted in the increased synthesis of ornithine-derived polyamines (Fig. 2g–i), which could additionally stimulate disease progression by participating in cancer cell proliferation [43] and tumour vascularization [44].

These changes were followed by alterations of ADMA synthesis regulating NOS-dependent function in the late phase of metastasis (Fig. 2f). The formation of a pre-metastatic niche is associated with early local pulmonary NO deficiency that contributes to lung metastasis [29–31] and systemic endothelial dysfunction linked to systemic inflammation [45, 46]. It could well be that ADMA-mediated impairment of NO production may also have a pathophysiological role in the development of systemic endothelial dysfunction associated with advanced cancer.

Altogether, we claim that induction of ARG activity detected in plasma in the early phase of metastasis formation in 4T1 metastatic breast cancer in mice contributes to metastasis progression. Indeed, it was shown that the ARG inhibitor *N*^ω^-hydroxy-nor-l-arginine reduced the inhibitory effect of MDSCs on T-cell proliferation and reduced the number and size of pulmonary metastasis [36]. Moreover, pegylated derivatives of ARG inhibited tumour cell growth by depletion of the arginine pool for polyamine synthesis [47]. Novel compounds reducing arginine activity are under investigation in humans: pegylated Arg1 AEB1102 in phase I/II study (NCT02488044) and ARG inhibitor CB-1158 in a phase I clinical trial (NCT02903914). Accordingly, changes in arginine metabolism detected in the plasma can represent an important and pathophysiologically relevant marker of early phases of cancer and metastasis.

Surprisingly, in contrast to changes in arginine metabolism that occurred in an early phase of metastasis, changes in energy metabolism (Fig. 4) were observed in the late phase of metastasis only and involved the switch of energy production to glycolysis (the ‘Warburg effect’), increased catabolism of Gln and accelerated PPP, all of which are characteristic of cancer development [48]. It might well be that the plasma glycolysis metabolome reflected not only a direct leakage of metabolites from tumour being big enough to induce changes in plasma metabolome, but also a significant recruitment and activation of immune cells in blood that also depends on glycolysis [49].

In the present work, we also identified a number of changes in lipid profile that all occurred in the late phases of cancer and metastasis (Fig. 5) and involved alterations related to structural and signalling lipid composition in blood plasma, which indicated remodelling of biological membranes and activation of lipid-dependent signals. In fact, changes in structural lipids such as increased formation of SM-rich lipids at the expense of PCs (Fig. 5a, i), reduction of lipid unsaturation (Fig. 5d), increased amount of ether lipids (Fig. 5f) and higher content of lipids with short acyl chains (Fig. 5f) may all contribute to alteration in membranes’ nanomechanical properties, to increased cancer aggressiveness [50, 51] and to increased vascular permeability [18]. In turn, increased release of many pro-proliferative and anti-apoptotic lipids, such as lysophosphatidic acid, derived from LysoPC (Fig. 5b) or platelet-activating factor, derived from ether phospholipids (Fig. 5d), can contribute to proliferation, survival, migration, vesicle trafficking and inflammation, tumourigenesis or metastasis through inter- and intra-cellular signalling [51–54], Interestingly, plasmalogens, also derived from ether lipids augmented at late metastatic stage, known to be important reservoirs of arachidonate, can activate Akt and extracellular signal-regulated kinase survival signalling, which protects against apoptosis [55]. The altered arachidonic acid metabolism reported here (Fig. 5g, h) is also a common feature of malignancies (*see* review [56]). In turn, the metabolism of SM leads to formation of ceramides and S1P, which are related to cancer cell proliferation and apoptosis regulation [57, 58]. There is good evidence supporting the hypothesis that S1P is involved in mesenchymal transformation, increased invasiveness, glucose metabolism or neovascularization [59]. Additionally, S1P is known as a regulator of endothelial barrier function [60] and therefore may influence the extravasation of cancer cells. In our study, transiently increased plasma S1P level (Fig. 5j) could contribute to early changes in endothelial permeability required for metastases formation.

In the present work, on the basis of multivariate statistical analysis, we confirmed that the plasma profile of arginine metabolism, energy metabolism and lipids was clearly distinct for 4T1 breast cancer-bearing mice and control mice, as well as for the late metastatic group (3 and 4 weeks after cancer cell inoculation) and the early metastatic and control groups (1–2 weeks after cancer cell inoculation), taken together. The early metastatic group was also to some extent differentiated from the control group on the basis of changes in arginine metabolism and some lipids. Our multivariate statistical analysis was performed based on ten animals per experimental groups. Obviously, this approach was sufficient for the detection of meaningful changes in targeted analysis of selected metabolites, but it was not sufficient to achieve high statistical power in multivariate statistical analysis.

## Conclusions

In the present work, we demonstrated that progression of 4T1 metastatic breast cancer in mice was linked to distinct metabolomic changes in plasma in the early and late phases of metastasis. The early phase of metastasis was featured by an increase in ARG activity and polyamine synthesis that can be associated with ARG-dependent immunosuppression and polyamine-dependent cellular proliferation, respectively. The late phase of metastasis was reflected in plasma by alterations in arginine pathways involving not only ARG activity and polyamine synthesis but also increased ADMA synthesis that can be linked with an impairment of endothelial function. However, the late phase of metastasis but not the early phase was featured in plasma by reprogramming of energy metabolism toward glycolysis and the pentose phosphate pathway at the expanse of the TCA, as well as by remodelling of structural and signalling lipids, all of which coincided with metastasis progression. Altogether, the ARG pathway constitutes an early plasma biomarker of cancer and metastasis and an important target for therapy, whereas metabolism reprogramming towards glycolysis and changes in signalling and structural lipids, if detected in plasma, constitute biomarkers for advanced cancer and the late phase of metastasis.

## Additional file

Additional file 1: **Table S1.** Gradient elution program applied for UPLC-MS analysis for positive ionisation mode. **Table S2.** Gradient elution program applied for UPLC-MS analysis for negative ionisation mode. **Table S3.** LC-MS/MS parameters in positive and negative ESI mode for determination of metabolites in plasma. **Table S4.** LC-MS/MS parameters for determination of S1P in plasma. (DOCX 24 kb)

## Abbreviations

ADMA: Asymmetric dimethylarginine
ANOVA: Analysis of variance
ARG: Arginase
DHAP: Dihydroxyacetone phosphate
E4P: Erythrose 4-phosphate
FIA: Flow injection analysis
GADP: D-glyceraldehyde 3-phosphate
Gln: Glutamine
Glu: Glutamate
iNOS: Inducible nitric oxide synthase
LysoPC: Lysophosphatidylocholines
MDSC: Myeloid-derived suppressor cells
NOS: Nitric oxide synthase
PC: Phosphatidylcholines
PLS-DA: Partial least squares discriminant analysis
PPP: Pentose phosphate pathway
S1P: Sphingosine-1-phosphate
SM: Sphingomyelins
TCA: Tricarboxylic cycle
