# Supplementary material for: Alterations in arginine and energy metabolism, structural and signalling lipids in metastatic breast cancer in mice detected in plasma by targeted metabolomics and lipidomics
Source: Breast Cancer Res. 2018 Dec 4;20:148. doi: 10.1186/s13058-018-1075-y (PMC6278167; doi:10.1186/s13058-018-1075-y)
Supplement: Supplementary file 1 — Table S1. Gradient elution program applied for UPLC-MS analysis for positive ionisation mode. Table S2. Gradient elution program applied for UPLC-MS analysis for negative ionisation mode. Table S3. LC-MS/MS parameters in positive and negative ESI mode for determination of metabolites in plasma. Table S4. LC-MS/MS parameters for determination of S1P in plasma. (DOCX 24 kb) [file 13058_2018_1075_MOESM1_ESM.docx]

Supplementary Materials and Methods

Alterations in Arginine and Energy Metabolism, Structural and Signalling Lipids in Metastatic Breast Cancer in Mice Detected in Plasma by Targeted Metabolomics and Lipidomics

Kamil Kus^1^, Agnieszka Kij^1,2^, Agnieszka Zakrzewska^1^, Agnieszka Jasztal^1^, Marta Stojak^1^,

Maria Walczak^1,2^, Stefan Chlopicki^1,3^

**The targeted metabolomics method**

Metabolites extraction procedure

An aliquot of 10 µL of plasma was used to extract the metabolites by addition of 0.5 mL of dry-ice-cold (-70°C) extraction mixture (acetonitrile: methanol: water 5:2:3, v/v/v). The extraction mixture was prepared at least 4-5 h before the analysis and placed in freezer. The samples were vortexed for 5 min and placed on dry ice for 30 min for protein precipitation. After that time samples were centrifuged at 15,000 x g, 4°C for 15 min. A volume of 250 µL of supernatant was lyophilized and dry extract were kept at -80°C until analysis.

LC/MS/MS conditions

The lyophilized samples were reconstituted in 50 µL of LC/MS/MS grade water and was injected onto LC/MS/MS column. A chromatographic studies were performed on a UFLC Nexera (Shimadzu, Kyoto, Japan). An Acquity UPLC BEH C18, 1.7 µm 3.0 x 100 mm (Waters, Milford, MA, USA) was employed as an analytical column. The samples were measured twice, injecting onto analytical column 5 µL of sample employing positive and 10 µL of sample employing negative ionisation mode. The gradient elution programs for both analysis are shown in Table S1 and S2.

For the detection QTRAP 5500 mass spectrometer (Sciex, Framingham, MA, USA) with a electrospray interfaced operated in positive and negative ionisation modes was used. The ion source operation conditions were: Curtain Gas: 25 psi, Collision Gas: medium, Temperature: 500°C, Ion Source Gas 1: 40 arb., Ion Source Gas 2: 50 arb. and IonSpray Voltage: 5500 V and -4500 V for positive and negative ionisation modes, respectively.

The LC/MS/MS method parameters for measured metabolites are summarized in Table S3.

Table S1. Gradient elution program applied for UPLC-MS analysis for positive ionisation mode.

| Time [min] | Flow [μL/min] | buffer A [%] | buffer B [%] |
| --- | --- | --- | --- |
| 0 | 0.25 | 3 | 97 |
| 0.5 |  | 3 | 97 |
| 2 |  | 15 | 85 |
| 5 |  | 70 | 30 |
| 5.5 |  | 70 | 30 |
| 6 |  | 3 | 97 |
| 8 |  | 3 | 97 |

Buffer A: ACN

Buffer B: 5 mM HCOONH4 (pH 5.8)

Table S2. Gradient elution program applied for UPLC-MS analysis for negative ionisation mode.

| Time [min] | Flow [μL/min] | buffer A [%] | Buffer B [%] |
| --- | --- | --- | --- |
| 0 | 0.25 | 2 | 98 |
| 1 |  | 2 | 98 |
| 2.5 |  | 95 | 5 |
| 3 |  | 95 | 5 |
| 3.5 |  | 2 | 98 |
| 6 |  | 2 | 98 |

Buffer A: ACN : 50 mM HCOONH4 (pH 5.8) (90:10, v/v)

Buffer B: 5 mM HCOONH4 (pH 5.8)

Table S3. LC/MS/MS parameters in Positive and Negative ESI Mode for determination of metabolites in plasma

| Positive ionisation | | | | | | | | |
| --- | --- | --- | --- | --- | --- | --- | --- | --- |
|  | q1 [m/z] | q3 [m/z] | CE [eV] | Retention Time [min] | LOD [pmol/mL] | LOQ [pmol/mL] | Linear range [pmol/mL] | r |
| Ethanolamine | 62.1 | 44.2 | 12 | 1.98 | 10 | 50 | 50-10000 | 0.998 |
| Glycine | 76 | 30.1 | 13 | 1.77 | 50 | 100 | 100-10000 | 0.9917 |
| Putrescine | 89 | 72 | 6 | 2.15 | 250 | 250 | 250-50000 | 0.992 |
| Alanine | 90.1 | 44.2 | 13 | 1.8 | 10 | 50 | 50-10000 | 0.9837 |
| GABA | 104.1 | 69.1 | 20 | 1.81 | 10 | 10 | 10-5000 | 0.9972 |
| Serine | 106 | 60 | 10 | 1.78 | 5 | 10 | 10-10000 | 0.9986 |
| Proline | 116.1 | 70.1 | 13 | 2.05 | 10 | 50 | 50-5000 | 0.9977 |
| Valine | 118.1 | 55.2 | 13 | 2.34 | 10 | 50 | 50-50000 | 0.9927 |
| Cysteine | 122 | 59.1 | 22 | 1.78 | 50 | 50 | 50-5000 | 0.9908 |
| Hydroxyproline | 132 | 68.2 | 19 | 1.81 | 10 | 10 | 10-2500 | 0.9846 |
| Isoleucine | 132.1 | 86 | 13 | 3.74 | 1 | 1 | 1-10000 | 0.9927 |
| Leucine | 132.1 | 86.01 | 13 | 4.02 | 1 | 5 | 5-10000 | 0.9957 |
| Ornithine | 133 | 70 | 14 | 1.84 | 5 | 10 | 10-5000 | 0.9932 |
| Asparagine | 133.1 | 74 | 19 | 1.78 | 10 | 50 | 50-10000 | 0.9896 |
| Aspartate | 134 | 74 | 17 | 1.63 | 10 | 50 | 50-10000 | 0.9831 |
| Homocysteine | 136.1 | 90.1 | 10 | 2.18 | 10 | 50 | 50-5000 | 0.9874 |
| Lysine | 147 | 67 | 32 | 1.86 | 10 | 50 | 50-5000 | 0.9845 |
| Glutamine | 147.1 | 84.1 | 30 | 1.81 | 10 | 10 | 10-10000 | 0.99 |
| Glutamate | 148.1 | 84.1 | 20 | 1.64 | 10 | 10 | 10-10000 | 0.9909 |
| Methionine | 150 | 133 | 12 | 3.05 | 10 | 50 | 50-10000 | 0.9913 |
| Homomethionine | 164 | 56 | 20 | 4.21 | 5 | 10 | 10-10000 | 0.992 |
| Phenylalanine | 166.1 | 103 | 30 | 4.91 | 1 | 10 | 10-10000 | 0.9962 |
| GADP_DHAP | 171 | 98.9 | 10 | 1.59 | 100 | 250 | 250-5000 | 0.9633 |
| Tryptophan | 205 | 146 | 18 | 5.21 | 1 | 5 | 5-5000 | 0.9959 |
| Cystathionine | 223 | 134 | 13 | 1.73 | 1 | 5 | 5-10000 | 0.9966 |
| Glucosamine-6-P | 259.9 | 126.1 | 20 | 1.68 | 10 | 10 | 10-10000 | 0.9905 |
| Hexose-P | 261 | 80.8 | 40 | 1.59 | 10 | 50 | 50-5000 | 0.9943 |
| GSH | 308 | 162 | 20 | 2.02 | 10 | 10 | 10-10000 | 0.9914 |
| SAH | 385.1 | 136 | 21 | 4.61 | 5 | 10 | 10-5000 | 0.9916 |
| SAM | 399.1 | 250 | 15 | 3.75 | 1 | 1 | 1-25000 | 0.9936 |
| Folate | 442 | 295 | 18 | 4.54 | 1 | 1 | 1-10000 | 0.9911 |
| 5-methyl-tetrahydrofolate | 460.1 | 313.1 | 21 | 4.66 | 10 | 50 | 50-10000 | 0.999 |
| GSSG | 613 | 231 | 35 | 1.64 | 1 | 10 | 10-10000 | 0.9971 |
| NAD | 663.9 | 135.8 | 40 | 4.31 | 5 | 5 | 5-5000 | 0.9919 |
| NADH | 666 | 648.7 | 20 | 4.34 | 50 | 50 | 50-5000 | 0.9914 |
| NADP | 744 | 135.9 | 45 | 2.47 | 1 | 5 | 5-5000 | 0.9971 |
| NADPH | 746 | 136 | 45 | 2.49 | 50 | 50 | 50-25000 | 0.9952 |
| N-acetyl-Glutamate | 190.1 | 84.1 | 24 | 1.57 | 10 | 50 | 50-25000 | 0.992 |
| Arginine | 175 | 60 | 16 | 2.00 | 1 | 1 | 1-10000 | 0.988 |
| Cystine | 241 | 74 | 28 | 1.73 | 10 | 10 | 10-10000 | 0.9918 |
| Negative ionisation | | | | | | | | |
|  | q1 [m/z] | q3 [m/z] | CE [eV] | Ret. Time [min] | LOD [pmol/mL] | LOD [pmol/mL] | Linear range [pmol/mL] | r |
| Pyruvate | 86.9 | 43 | -15 | 1.55 | 10 | 50 | 50-50000 | 0.9892 |
| Lactate | 88.9 | 43 | -20 | 1.71 | 5 | 50 | 50-10000 | 0.98 |
| Fumarate | 114.9 | 71 | -15 | 1.53 | 10 | 50 | 50-50000 | 0.9898 |
| Succinate | 117.1 | 73.3 | -20 | 1.60 | 5 | 50 | 50-10000 | 0.9946 |
| alpha-Ketoglutarate | 144.9 | 101 | -10 | 1.57 | 10 | 10 | 10-50000 | 0.9971 |
| Glutamine | 145.1 | 127.2 | -10 | 1.82 | 1 | 1 | 1-10000 | 0.9937 |
| Glutamate | 146.1 | 128 | -15 | 1.65 | 5 | 5 | 5-10000 | 0.9753 |
| alpha-Hydroxyglutarate (2-HG) | 146.9 | 128.9 | -15 | 1.53 | 5 | 10 | 10-1000 | 0.9899 |
| GAHP_DHAP | 168.9 | 78.9 | -40 | 1.60 | 5 | 10 | 10-5000 | 0.9805 |
| Hexose | 179 | 113 | -15 | 1.87 | 1 | 5 | 5-10000 | 0.9787 |
| N-acetylglutamate | 188.1 | 128.1 | -15 | 1.58 | 10 | 10 | 10-50000 | 0.9929 |
| Citrate | 191.1 | 87.1 | -30 | 1.65 | 10 | 50 | 50-50000 | 0.9942 |
| Isocitrate | 191.1 | 117.2 | -20 | 1.62 | 1 | 1 | 1-10000 | 0.9906 |
| E-4-P | 198.9 | 96.8 | -20 | 1.67 | 5 | 10 | 10-10000 | 0.9904 |
| Pentose-5-P | 228.91 | 78.9 | -40 | 1.64 | 1 | 1 | 1-50000 | 0.9914 |
| Glucosamine-6-P | 257.9 | 96.9 | -20 | 1.73 | 10 | 10 | 10-10000 | 0.9928 |
| Hexose-P_1 | 259 | 78.9 | -40 | 1.59 | 10 | 10 | 10-10000 | 0.9877 |
| Hexose-P_2 | 259 | 97 | -10 | 1.59 | 1 | 5 | 5-10000 | 0.9888 |
| 6-P-Gluconate | 274.9 | 256.8 | -15 | 1.49 | 50 | 250 | 250-50000 | 0.9909 |
| S-7-P | 288.9 | 96.8 | -40 | 1.58 | 5 | 5 | 5-5000 | 0.9906 |
| GSH | 306 | 254 | -25 | 1.67 | 10 | 10 | 10-25000 | 0.995 |
| Fructose-1,6-biP | 339 | 97 | -30 | 1.45 | 1 | 1 | 1-50000 | 0.9927 |
| SAH | 383.1 | 134 | -30 | 4.16 | 1 | 1 | 1-5000 | 0.9991 |
| GSSG | 611 | 305.9 | -25 | 1.67 | 1 | 1 | 1-25000 | 0.9902 |
| NAD | 661.9 | 540 | -20 | 4.17 | 1 | 1 | 1-5000 | 0.9932 |
| NADH | 664 | 408 | -31 | 4.16 | 10 | 50 | 50-1000 | 0.9939 |
| NADP | 741.9 | 620 | -30 | 2.95 | 10 | 10 | 10-25000 | 0.9924 |
| NADPH | 743.9 | 408 | -34 | 2.95 | 10 | 50 | 50-10000 | 0.9994 |

LOD – limit of detection

LOQ – limit of quantification

r – correlation coefficient

**The method for determination of sphingosine-1-phosphate (S1P) by LC/MS/MS**

Sample preparation

Ten μL of plasma was spiked with 10 μL of internal standard - D-erytro-C17 (reaching the final concentration of IS 1 uM in each sample) and deprotonated by the addition of 500 μL of acetonitrile, vortex vigorously for 1 min and placed on ice for 10 min. After that time, samples were centrifuged at 15,000 x g, 4°C for 15 min and 5 μL of supernatant was injected onto LC column.

LC/MS/MS conditions

The concentration of S1P was measured by an LC/MS/MS technique using a TSQ Quantum mass spectrometer system (Thermo Scientific, Waltham, MA, USA) coupled to a UHPLC UltiMate 3000 (Dionex, Sunnyvail, CA, USA). Chromatographic separation was achieved with a Hypersil GOLD, 1.9 µm, 2.1 mm x 50 mm (Thermo Scientific, Waltham, MA, USA) analytical column using acetonitrile and water, both with a 0.1% (v/v) of formic acid addition as mobile phase under isocratic condition (40% acetonitrile) at a flow rate of 0.8 mL/min. The working parameters of mass spectrometer were as follows: Ion Spray Voltage: 3500 V, Vaporizer Temperature: 300°C, Sheath Gas Pressure: 40 arb., Auxiliary Gas Pressure: 25 arb., Capillary Temperature: 300°C, Collision Pressure: 1.5 mTorr. Argon was used as collision gas. The LC/MS/MS conditions for S1P and IS were summarized in Table S4.

Table S4. LC/MS/MS parameters for determination of S1P in plasma

|  | q1 [m/z] | q3 [m/z] | CE [eV] | Retention Time [min] | LOD [µM] | LOD [µM] | Linear range [µM] | r |
| --- | --- | --- | --- | --- | --- | --- | --- | --- |
| D-erytro-C17 (IS) | 286.2 | 268.2 | 7 | 0.46 | - | - | - | - |
| S1P | 380.2 | 264.2 | 14 | 0.45 | 0.01 | 0.05 | 0.05-50 | 0.9919 |
